# Reliance on social security benefits by Swedish patients with ill-health attributed to dental fillings: a register-based cohort study

**DOI:** 10.1186/1471-2458-12-713

**Published:** 2012-08-30

**Authors:** Aron Naimi-Akbar, Pia Svedberg, Kristina Alexanderson, Jan Ekstrand, Gunilla Sandborgh-Englund

**Affiliations:** 1 Division of Dental Biomaterials and Cariology, Department of Dental Medicine, Karolinska Institutet, Stockholm, Sweden; 2 Division of Insurance Medicine, Department of Clinical Neuroscience, Karolinska Institutet, Stockholm, Sweden; 3 Clinical Epidemiology Unit, Department of Medicine, Karolinska Institutet, Stockholm, Sweden

**Keywords:** Dental materials, Dental amalgam, Sick leave, Social security, Work, Register based

## Abstract

**Background:**

Some people attribute their ill health to dental filling materials, experiencing a variety of symptoms. Yet, it is not known if they continue to financially support themselves by work or become reliant on different types of social security benefits. The aim of this study was to analyse reliance on different forms of social security benefits by patients who attribute their poor health to dental filling materials.

**Methods:**

A longitudinal cohort study with a 13-year follow up. The subjects included were 505 patients attributing their ill health to dental restorative materials, who applied for subsidised filling replacement. They were compared to a cohort of matched controls representing the general population (three controls per patient). Annual individual data on disability pension, sick leave, unemployment benefits, and socio-demographic factors was obtained from Statistics Sweden. Generalized estimating equations were used to test for differences between cohorts in number of days on different types of social security benefits.

**Results:**

The cohort of dental filling patients had a significantly higher number of days on sick leave and disability pension than the general population. The test of an overall interaction effect between time and cohort showed a significant difference between the two cohorts regarding both sick leave and disability pension. In the replacement cohort, the highest number of sick-leave days was recorded in the year they applied for subsidised replacement of fillings. While sick leave decreased following the year of application, the number of days on disability pension increased and peaked at the end of follow-up.

**Conclusions:**

Ill health related to dental materials is likely to be associated with dependence on social security benefits. Dental filling replacement does not seem to improve workforce participation.

## Background

Epidemiological research has shown that the probability of poor health in people with amalgam fillings is not higher than among others [1-3]. However, some individuals attribute their ill health to dental filling materials; they experience a variety of symptoms such as fatigue, sleep disturbance, and joint and muscle pain [4-6]. It is not known whether these individuals continue to financially support themselves by work or become reliant on different types of social security benefits.

Since 1999, the Swedish National Dental Health Insurance Scheme has included subsidised replacement of dental fillings to relieve symptoms allegedly due to adverse effects of dental filling materials [7]. The eligibility criteria, according to the Act and the Ordinance on State Dental Care, include severe symptoms not due to other causes, identified through a thorough medical investigation and inclusion of dental filling replacement in an overall treatment plan, under the guidance of a physician with specialist training in a field relating to the patient’s symptoms [7].

Several clinical trials have shown improvement in self-rated health in this patient group following dental filling replacement [8-11]. However, it has been questioned whether the improvement is actually an effect of the dental filling replacement [10,11]. Moreover, no studies have addressed the question of whether the replacement of fillings leads to improved work participation.

The aim of this study was to examine social security benefits data for patients with health symptoms related to dental materials, with special reference to such factors as prior and subsequent reliance on sick leave, disability pension, unemployment benefits, and early old-age pension. Applicants for subsidised replacement of dental fillings were compared with a representative cohort from the general population. A further comparison was made between successful applicants for subsidised replacement of dental fillings and those whose application was rejected.

## Methods

### Study design, participants, and setting

A cohort of patients under the age of 65 suffering from ill health which they attributed to their dental filling materials was followed with regard to their use of social security benefits between 1994 and 2006. This cohort was compared with a cohort of matched individuals representing the general population followed over the same years. For each patient, three people from the general population were matched with respect to sex, age, and county of residency.

The cohort of patients with health symptoms related to dental materials comprised all applicants for subsidised dental filling replacement between 1999 and 2005, *i.e.* all 505 applicants under the age of 65 in the year of the application. All 21 Swedish counties were asked to provide patient information for the study. Seven counties, representing 39% of the Swedish population agreed to contribute and provided patient information. Applications for subsidised filling replacement are processed at county level. The subjects were classified into two groups, according to whether their application for subsidised replacement of fillings had been approved (fully or partly) or rejected. Those who had applied more than once were classified into the approval group if at least one application had been approved. The procedure of filling replacement is performed as a common dental procedure, conducted by a dentist. The fillings are replaced by a dental materials considered as less perilous by the patient, e.g. plastic composite materials.

### Social insurance in Sweden

All people living in Sweden are covered by the national social insurance scheme. All adult residents with income from work or unemployment benefits can get sickness benefits covering up to 80% of lost income when unable to work due to disease or injury. The first sick-leave day is a qualifying day with no benefits. After the 7th sick-leave day, a sickness certificate issued by a physician is required. The employer provides sick pay for the first 14 days of a sick-leave spell and thereafter, i.e. from day 15, the sickness benefits are provided by the Social Insurance Agency. In this study we have data of the latter, and not of the shorter sick-leave spells paid by the employer.

Individuals with a medically confirmed disease or injury that permanently restricts their work capacity can be granted a disability pension, covering at least 65% of lost income. The general old-age retirement age is 65 years but can be taken earlier.

### Data collection

In 2008, background information on applicants for subsidised dental filling replacement was collected from the seven county councils, including sex, date of birth, the number and date of the subsidised dental filling replacement applications, as well as the resultant decision on the application: fully approved, partly approved, or rejected.

Based on these data, Statistics Sweden constructed the general population cohort through matches with the Total Population Register of all residents of Sweden, according to the above described procedure, with a matching ration of 1:3 (n = 1496).

For both cohorts, individual information about educational level, other socio-demographic factors, as well as different types of income, social welfare benefits and the number of days on unemployment benefits, sick leave, and disability pension was obtained from Statistics Sweden for each of the years 1994 to 2006, through linkage to the Longitudinal integration database for health insurance and labour market studies (LISA), using the Swedish personal identity number [12].

### Outcome variables

The outcome measures were the annual number of days on disability pension, sick leave, and unemployment benefits, respectively. Income data were retrieved as the total income in Swedish kronor for each subject each year. Receiving social welfare benefits or old-age pension during a year was categorised as yes or no.

### Background variables

Education was stratified into three levels, according to the highest level attained (Table 1). Four categories were used for country of birth: Sweden; other Nordic countries, other European countries, or outside Europe. Year of birth was sorted into decades (1920’s, 1930’s, etc.). In the replacement cohort, age at year of application was calculated and used as a continuous variable.

**Table 1:** Sociodemographic characteristics of the participants and year of application in the replacement cohort

	**Replacement cohort**				**General population cohort**	
	**All**		**Approved**			
	**N=505**		**N=322**		**N=1496**	
**Decade of birth**						
1930's	32	6.3%	21	6.5%	96	6.4%
1940's	175	34.7%	115	35.7%	522	34.9%
1950's	172	34.1%	112	34.8%	504	33.7%
1960's	109	21.6%	66	20.5%	324	21.7%
1970's	16	3.2%	8	2.5%	47	3.1%
1980's	1	0.2%	0	0.0%	3	0.2%
**Sex**						
Female	404	80.0%	269	83.5%	1194	79.8%
Male	101	20.0%	53	16.5%	302	20.2%
**Educational level**						
Primary and lower secondary school	70	13.9%	46	14.3%	303	20.3%
Upper secondary school	240	47.5%	161	50.0%	663	44.3%
Post-secondary school	193	38.2%	113	35.1%	524	35.0%
Data unavailable	2	0.4%	2	0.6%	6	0.4%
**Country of birth**						
Sweden	422	83.6%	280	87.0%	1257	84.0%
Other Nordic countries	35	6.9%	20	6.2%	105	7.0%
Other European countries	26	5.1%	13	4.0%	69	4.6%
Outside Europe	22	4.4%	9	2.8%	65	4.3%
**Application year**						
1999	49	9.7%	29	9.0%		
2000	77	15.2%	47	14.6%		
2001	97	19.2%	68	21.1%		
2002	77	15.2%	48	14.9%		
2003	96	19.0%	66	20.5%		
2004	54	10.7%	28	8.7%		
2005	55	10.9%	36	11.2%		

### Statistics

Proportional differences in background variables were tested using the chi^2^-test.

Days on disability pension, sickness benefits, and unemployment benefits and annual income were analysed using generalized estimating equations (GEE) [13]. Annual (1994 to 2006) number of days on disability pension, sickness benefits, and unemployment benefits and income were modeled as a series of repeated measures. We used the assumption of the Poisson distribution of the dependent variable, with an autoregressive correlation structure and used the log link function. The model included cohort (replacement/general population), year modeled as a series of dichotomised dummy variables, and an interaction term between year and cohort. Analyses were adjusted for educational level, country of birth, and sex as dichotomous dummy variables, and age as a continuous variable.

Early old-age pension and social welfare benefits (yes or no) were also analysed with GEE, but assumed the binomial distribution and the logit link function.

The analyses were performed within the replacement cohort in relation to the application year, that is, the year of application (T_0_), and included the previous five years as well as the subsequent five years. Differences in days on disability pension and sickness benefits between those whose applications had been approved or rejected, respectively,were analysed using GEE. We used the assumption of the Poisson distribution of the dependent variable, with an autoregressive correlation structure and used the log link function. The model included application approval (approval/rejection), year modeled as a series of dichotomised dummy variables and an interaction term between year and application approval. Analyses were controlled for educational level, country of birth, and sex as dichotomous dummy variables, and age as a continuous variable.

Subjects were included in the analyses up to the year they turned 65. Subjects who emigrated were not included following the year of emigration.

IBM SPSS Statistics 20 and STATA version 12 was used for statistical analyses. P-values less than 0.05 were considered as statistically significant.

### Ethics

Ethical approval was granted from the Regional Ethical Review Board in Stockholm, Sweden.

## Results

The cohort characteristics are presented in Table 1. Comparison of the replacement cohort with the matched general population cohort disclosed no demographic differences, except for educational level – a higher percentage of the general population cohort belonged to the lowest educational level: 20.3%, compared with 13.9% of the replacement cohort (p = 0.001). Within the replacement cohort, the proportion of women was somewhat higher among the approved than among the rejected applicants: 83.5 and 73.8% respectively (p = 0.008).

Of the 505 subjects in the replacement cohort, subsidised filling replacement was approved for 322 (63.8%). For T_-5,_ that is_,_ five years before the application year, data from LISA were available for 319 (99.1%) of the approved group and 180 (98.4%) of the rejection group. The corresponding values for the year of application were 321 (99.8%) for the approved group (one subject in the approved group had died during the application year and thus not included in LISA) and 183 (100.0%) for the rejected group.

### Replacement cohort vs. general population cohort

**Figure 1:**
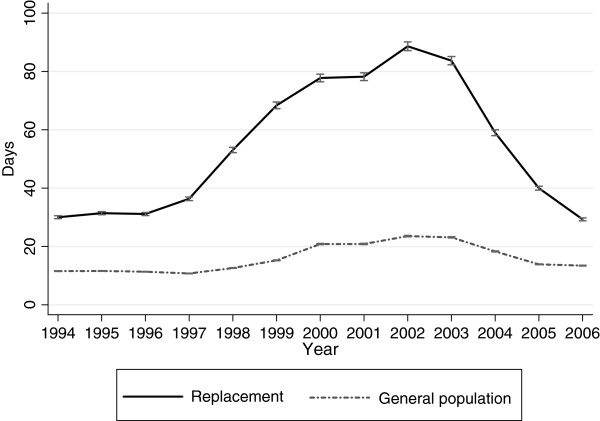
**Sick-leave in the replacement cohort and the general population cohort.** Annual days on sick-leave in the replacement cohort and the general population cohort in 1994–2006. Predicted averages with 95% confidence intervals derived from regression analyses with generalized estimating equations.

Figure 1 displays the predicted annual averages of sick-leave days in 1994 to 2006 for both cohorts. The pattern of changes showed an increased difference between the two cohorts until 2002 when it begun to decrease until the last year of follow-up. There was a significant association between being in the replacement cohort and an increase in sick-leave days (p <0.001) (Table 2). The test of an overall interaction effect between time (year) and cohort showed a significant difference between the two cohorts (p <0.001).

**Table 2:** Results of generalized estimating equations models for sick leave and disability pension

	**Sick leave**		**Disability pension**	
	**β**	**SE**	**β**	**SE**
**Cohort**				
General population (ref)				
Replacement cohort	0.9673	0.0113	0.9530	0.0097
**Year**				
1994 (ref)				
1995	0.0037	0.0067	0.0695	0.0022
1996	−0.0182	0.0085	0.2017	0.0030
1997	−0.0730	0.0096	0.3313	0.0036
1998	0.0883	0.0098	0.4145	0.0040
1999	0.2767	0.0097	0.5012	0.0044
2000	0.5901	0.0093	0.5971	0.0047
2001	0.5909	0.0094	0.6981	0.0049
2002	0.7128	0.0093	0.8105	0.0051
2003	0.6948	0.0094	0.8825	0.0053
2004	0.4602	0.0099	1.0004	0.0055
2005	0.1832	0.0106	1.1193	0.0056
2006	0.1508	0.0107	1.1505	0.0058
Cohort*Year				
1994 (ref)				
1995	0.0415	0.0097	0.0915	0.0032
1996	0.0545	0.0124	0.1181	0.0044
1997	0.2641	0.0136	0.1052	0.0053
1998	0.4815	0.0136	0.1453	0.0059
1999	0.5463	0.0135	0.2163	0.0065
2000	0.3616	0.0133	0.2760	0.0069
2001	0.3664	0.0134	0.3490	0.0073
2002	0.3696	0.0133	0.3620	0.0076
2003	0.3306	0.0134	0.4090	0.0079
2004	0.2150	0.0142	0.4426	0.0081
2005	0.1030	0.0153	0.4239	0.0083
2006	−0.1757	0.0161	0.4046	0.0085
**Sex**				
Female	0.2813	0.0066	0.2814	0.0069
Male (ref)				
**Educational level**				
Primary and lower secondary school (ref)				
Upper secondary school	−0.0922	0.0064	−0.2034	0.0061
Post-secondary school	−0.3352	0.0070	−0.7667	0.0072
Data unavailable	−1.1064	0.0631	−0.0438	0.0342
**Country of birth**				
Sweden (ref)				
Other Nordic countries	0.1981	0.0087	0.0478	0.0095
Other European countries	0.1873	0.0102	0.3582	0.0098
Outside Europe	−0.0898	0.0129	0.2500	0.0124
**Age**	0.0004	0.0003	0.0305	0.0003
**Constant**	2.0472	0.0209	0.8424	0.0223

Regression coefficients with standard errors with sick leave and disability pension as dependent variables and cohort, year, cohort* year (interaction), educational level, country of birth, sex, and age also included in the model.

**Figure 2:**
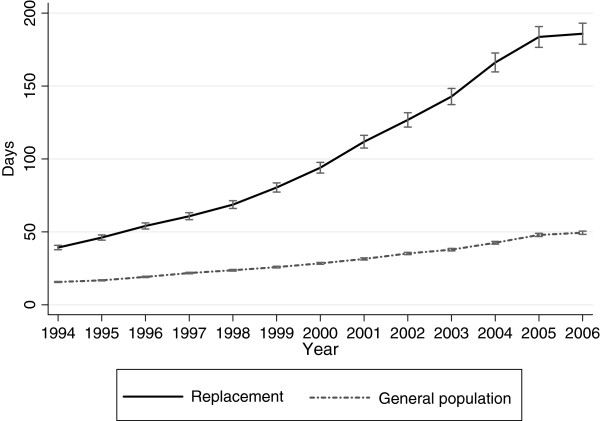
**Disability pension in the replacement cohort and the general population cohort.** Annual days on disability pension in the replacement cohort and the general population cohort in 1994–2006. Predicted averages with 95% confidence intervals derived from regression analyses with generalized estimating equations.

Figure 2 displays the predicted annual averages of days on disability pension 1994 to 2006; there was a steeper increase in the replacement cohort (p <0.001) (Table 2). The test of an overall interaction effect between time and cohort showed a significant difference between the two cohorts regarding the number of days on disability pension (p <0.001).

**Figure 3:**
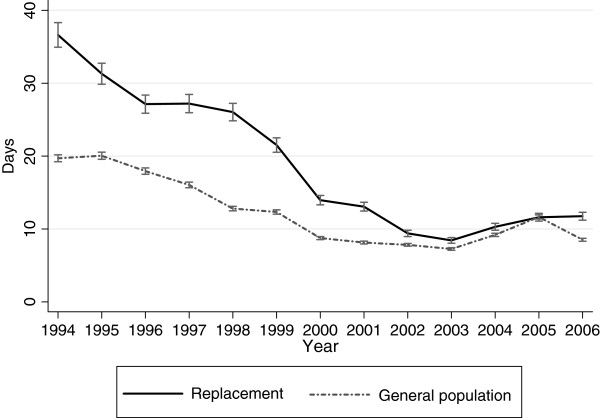
**Unemployment benefits in the replacement cohort and the general population cohort.** Annual days on unemployment benefits in the replacement cohort and general population cohort in 1994–2006. Predicted averages with 95% confidence intervals derived from regression analyses with generalized estimating equations.

The difference in days on unemployment benefits was highest early in the follow-up and decreased over time (Figure 3). The interaction between cohort and time was significant (p <0.001).

**Figure 4:**
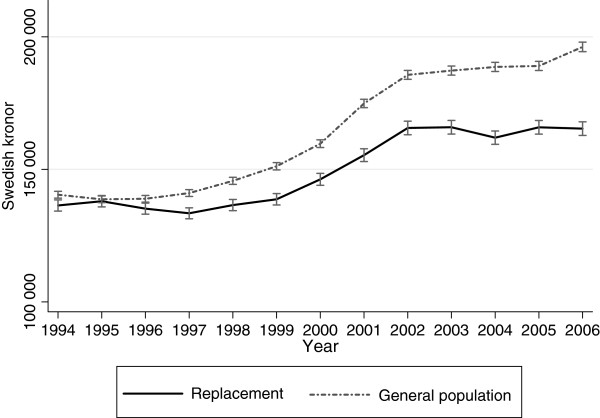
**Income in the replacement cohort and the general population cohort.** Annual income in Swedish kronor (SEK) in the replacement cohort and the general population cohort in 1994–2006. Predicted averages with 95% confidence intervals derived from regression analyses with generalized estimating equations.

As shown in Figure 4, the income differences between the two cohorts increased between 1994 and 2006. There was a significant interaction effect between cohort and time (p <0.001).

**Figure 5:**
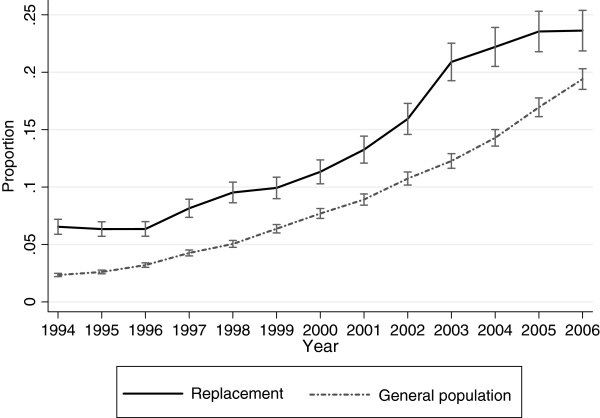
**Old-age pension in the replacement cohort and the general population cohort.** Proportions with early old-age pension in the replacement cohort and general population cohort in 1994–2006. Predicted proportions with 95% confidence intervals derived from regression analyses with generalized estimating equations.

**Figure 6:**
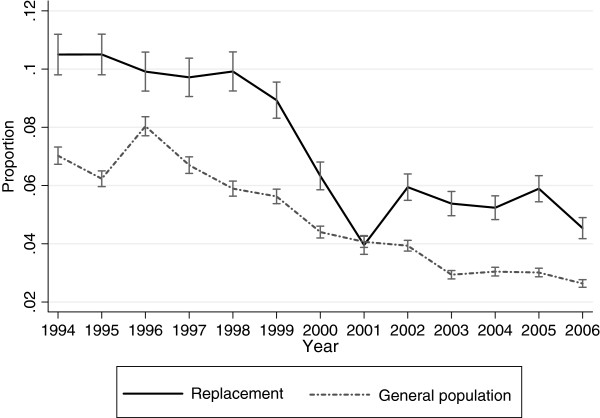
**Social welfare support in the replacement cohort and the general population cohort.** Proportions depending on social welfare support in the replacement cohort and the general population cohort in 1994–2006. Predicted proportions with 95% confidence intervals derived from regression analyses with generalized estimating equations.

The proportions taking early old-age pension was significantly higher in the replacement cohort at baseline (p <0.001) but there was no interaction between cohort and time (p > 0.05) (Figure 5). The proportion depending on social welfare benefits was significantly higher in the replacement cohort at baseline (p <0.01), but there was a no interaction between time and cohort (p > 0.05) (Figure 6).

### Approval vs. Rejection

**Figure 7:**
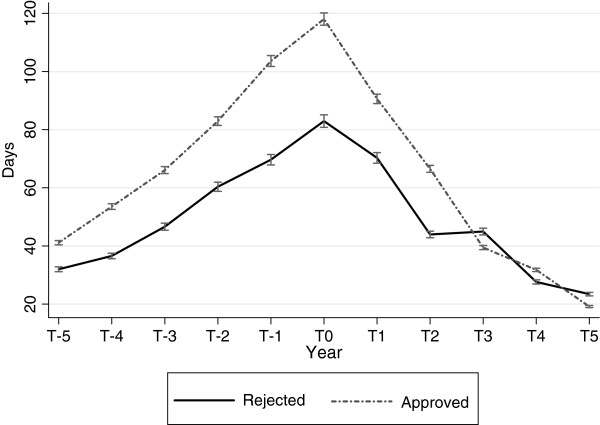
**Sick-leave in the replacement cohort among approved and rejected applicants.** Annual sick-leave days in the replacement cohort among approved and rejected applicants, five years before and after application for replacement of dental fillings. Predicted averages with 95% confidence intervals derived from regression analyses with generalized estimating equations. T_0_ is the year the applicant submitted the application.

Figure 7 presents the annual predicted average number of sick-leave days for the two groups of applicants, ‘approved’ and ‘rejected’, in the replacement cohort: T_0_ is the year of the application for subsidised filling replacement. Days on sick leave increased in both groups up to T_0,_ but was lower in the rejected group (p <0.001).

**Figure 8:**
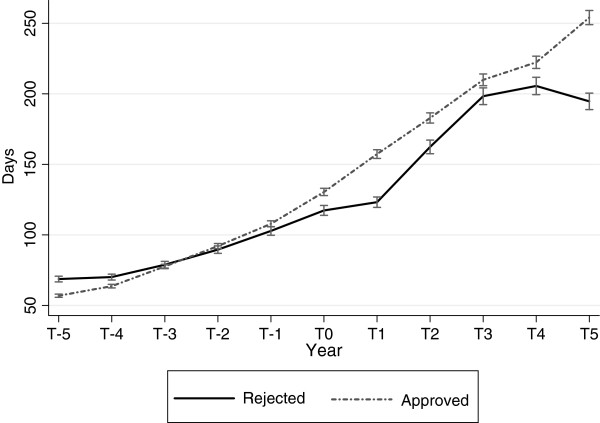
**Disability pension in the replacement cohort among approved and rejected applicants.** Annual sick-leave days in the replacement cohort among approved and rejected applicants. Predicted averages with 95% confidence intervals derived from regression analyses with generalized estimating equations. T_0_ is the year the applicant submitted the application.

Figure 8 shows days on disability pension for the two groups of applicants. The approved group had a lower baseline average (p <0.001) but their number of days increased at a higher pace compared to the rejected group, as shown by a significant interaction effect between time and group (p <0.001)

## Discussion

As highlighted by The Swedish Council on Technology Assessment in Health Care (SBU) [14], little is known about reliance on sick leave and disability benefits by different patient groups. The present study is the first to investigate reliance on social security benefits by individuals who attribute their ill health to dental materials. Comparison of a cohort applying for subsidised dental filling replacement with a cohort from the general population, disclosed much greater reliance by the replacement cohort on sick leave and disability pensions. This was the case for all thirteen years studied, even after replacement of dental fillings.

The study also compared outcomes for applicants who received approval for subsidised replacement of dental filling materials and those whose applications were rejected. Presuming that the approved group would have had their dental fillings replaced, in contrast to the rejected group, we aimed to study if there were any differences between the groups in terms of reliance on different forms of social security benefits the time after the application. Compared with those whose applications were rejected, approved applicants showed greater absence from work on sick leave in the years before and during the year of application. In the fifth year after filling replacement, significantly more patients in this group were receiving disability pensions.

After T_0_, the year of application, the number of sick-leave days decreased dramatically in the replacement cohort. However, the number of days absent from work did not change, because the days on disability pension increased rapidly in the same years. This rapid shift from sick leave to disability pension following filling replacement is in accordance with the results of a Swedish study of long-term sickness absentees who were referred to multidisciplinary medical assessment by the National Social Insurance Agency [15]. The same pattern emerges: neither multidisciplinary medical assessment nor dental filling replacement improves the likelihood of a return to work but rather enhances the path towards disability pension. Our results corroborate the difficulties associated with helping patients on long-term sick leave to resume workforce participation.

The level of education in the replacement cohort was somewhat higher than in the general population cohort, but this was not reflected in a correspondingly higher income. It has been shown that absence from work might lead to fewer promotions and lower salary [16,17]. However, in the present study, the results might also reflect the lower income from social insurance benefits compared to work salary. Our study included more women than men, which is in agreement with other studies concerning patients with general health problems related to dental materials [4,5,8,10]. This coincides with reports of higher prevalence of chronic pain conditions among women [18]. Several different potential causes have been suggested such as the impact of domestic responsibilities and job strain among women and a higher degree of catastrophizing among women [19,20].

The major strengths of this study are the large sample size and the possibility of comparing a patient cohort with one from the general population with three matched controls per patient. Further strengths are: the prospective study design, with not only long follow-up but also that data about the situation before inclusion into the cohort was available; no loss of subjects to follow-up, access to high-quality registry data, free from recall bias, and that we could include a large variety of types of incomes and benefits. Classification of patients with health problems related to dental restorative materials is always problematic as this is a very heterogeneous group, with a variety of health problems. This study population included all applicants for subsidised dental filling replacement in almost half of Sweden; this particular means of sampling reduces the risk of misclassification.

Limitations include that we have no information about whether filling replacement actually was done, e.g. in the rejection group. However, there were probably several individuals in the rejected group who paid themselves for having their fillings replaced. The differences between approval and rejection in the replacement cohort indicate that the general health in the approved group was poorer than in the rejected group. Rejection of an application was presumably based on failure to meet all the criteria for subsidised filling replacement. Another limitation is the unknown external validity for individuals in the non-participating counties.

Health risks associated with dental fillings primarily concern mercury exposure from dental amalgam fillings. In some clinical trials of patients with symptoms attributed to dental filling materials, improvement in self-rated health has been reported after dental filling replacement [10,11]. However, interventions other than filling removal have resulted in the same level of improvement in self-rated health [11]; thus, it is questionable whether the reported improvements could in fact be attributable to the removal of filling materials. The results of the present study indicate that the potential improvements in self-rated health following filling replacement are not enough to improve the patients’ work capacity. On the contrary, in the years succeeding filling replacement, a large proportion of the subjects were on sick leave or even on disability pension.

It has been suggested that the health problems these patients experience, rather than being a toxicological effect of mercury exposure or other dental filling materials, are attributable to a tendency to somatisation and that negative life events strongly influence the risk of impaired health related to dental amalgam [21-23]. These questions were beyond the aims of the present study. However, the results indicate that this group tends to be marginalised from the labour market. This might be prevented by earlier intervention.

## Conclusions

Ill health related to dental materials is likely to be associated with dependence on social security benefits. Dental filling replacement does not seem to improve workforce participation. The results indicate that further investigation is warranted to find ways of supporting this group of patients in returning to the workforce.

## Competing interests

The authors declare that they have no competing interests.

## Authors’ contributions

All authors contributed to the study design. ANA, PS, KA, and GSE participated in the data collection. ANA undertook the statistical analyses and was responsible for writing the first draft of the manuscript. ANA, PS, KA, and GSE were involved in interpreting the results. JE critically revised the manuscript. All authors read and approved the final manuscript.

## Pre-publication history

The pre-publication history for this paper can be accessed here:

http://www.biomedcentral.com/1471-2458/12/713/prepub
